# Supplementary material for: Adverse childhood experiences (ACEs) associated with reduced cognitive flexibility in both college and community samples
Source: PLoS One. 2021 Dec 2;16(12):e0260822. doi: 10.1371/journal.pone.0260822 (PMC8638954; doi:10.1371/journal.pone.0260822)
Supplement: Attachment — Submitted filename: Revision-Letter.docx [file pone.0260822.s001.docx]

PONE-D-21-09458
Adverse childhood experiences (ACEs) associated with reduced cognitive flexibility in both college students and adults

Revised title: ***Adverse childhood experiences (ACEs) associated with reduced cognitive flexibility in both college and community samples***

*PLOS ONE*

Dear Dr. Klengel,

Thank you for providing us with this opportunity to revise and resubmit our manuscript. We appreciate that you saw merit in our work. We are grateful to the reviewers’ for their thoughtful comments. We were pleased to read that they found our work interesting and applicable. The feedback we received was very helpful, and we believe it has substantially improved the quality of the manuscript.

We appreciate the opportunity to respond to the comments and suggestions made by the reviewers. Please find attached our revised manuscript to be reconsidered for publication at *PLOS ONE.* Changes made to the manuscript based on reviewer feedback is in blue ink.

As indicated below, this revised manuscript takes into account all of the issues raised by the reviewers. Reviewer comments are in plain text and our replies are in ***bold and italicized***.

Reviewer 1:

1. Last sentence reads reduced flexibility in adulthood. But, the results only focus on reduced cognitive flexibility.

***We thank the reviewer for this comment and agree with them. The language in the line has been amended to clarify that our results only apply to cognitive flexibility. Please see page 2.***

2. The authors should consider introducing ACEs in a slightly different way to encompass what ACEs are more fully. Instead of saying: “…humans who do not get the parental attention they need in early development” they could say: “humans who do not get basic needs met in early development”, as ACEs can consist of many things aside from concerns pertaining to parental attention

***We thank the reviewer for this suggestion. Language in introduction has been amended as recommended. See page 3.***

3. For the paragraph pertaining to flexibility: the author could benefit from briefly defining both cognitive and behavioral flexibility before providing the elaboration on how cognitive flexibility impacts behavioral flexibility.

***We thank the reviewer for this suggestion! We have added language briefly defining cognitive flexibility and behavioral flexibility within the perspective of this manuscript, as well as an elaboration of how the relations between the concepts have been described. See page 3.***

4. For the Harms study, what was ELA measured by? How much words did the ELA group perform than controls? Was it meaningful?

***We thank the reviewer for these important questions. Information discussing the measurement of ELA in the Harms study has been added to the Introduction (See page 5). With regard to whether the difference in performance between the ELA and control groups was meaningful; unfortunately Harms and colleagues did not provide descriptive statistics of the group differences in performance. However, the differences remained significant after accounting for family SES. We have reported this in the manuscript. Additionally, we were able to use the f-statistics reported by Harms et al to calculate the partial eta squared as a measure of effect size for the relevant analyses. The partial eta squared statistic estimates the ratio of variance in the outcomes (instrumental learning and cognitive flexibility) accounted for by the group (ELA v. Control) in proportion to the variance accounted for by the group and error. The partial eta squared statistics for group differences in performance on the instrumental learning and cognitive flexibility tasks ranged from .0996 to .2111, suggesting that the effects of group on measures of instrumental learning and cognitive flexibility were in the medium to large range.***

5. In the Kalia and Knauft study, participants only performed poorly on the CFI-control. What is the other aspect assessed on the CFI? Why might participants have performed “fine” on that?

***We thank the reviewer for these pertinent questions. The other aspect of the CFI that was assessed was CFI-Alternatives, or the ability to generate multiple solutions, perspectives, or explanations under stress or when faced with a difficult situation. In the Kalia and Knauft study, no association was found between ACEs and CFI-Alternatives. Language has been added page # clarifying the two CFI subscales and their associations with ACEs as reported in the Kalia and Knauft study. We appreciate the reviewer’s question about the link between CFI-Alternatives and ACEs. CFI-Alternatives was not correlated with the reported number of ACEs (r = -.04). This suggests that CFI-Alternatives did not change linearly with increases in ACEs. Since ACEs were measured continuously, this doesn’t allow us to comment on the overall performance of all with ACEs compared to all without. Though CFI-Control and Alternatives have been found to be positively correlated, they frequently differ in their association with other variables (Dennis & Vander Wal, 2010). Furthermore, there is evidence that stress can have unique impacts on cognitive flexibility depending on the aspect of cognitive flexibility being measured (Goldfarb et al., 2017; Kalia et al., 2018). We have added some language in the introduction to clarify this. See page 6.***

6. Please clarify inclusion and exclusion criteria for each sample

***We thank the reviewer for this comment. We have added language to clarify the inclusion criteria and exclusion criteria for the studies. Because Study 1 was completed in the laboratory and Study 2 was conducted online, the participant inclusion and exclusion criteria are slightly different for the two studies. See pages 8 & 11.***

7. Please clarify if participants in their second sample (adults) excluded adults currently attending college

***We thank the reviewer for this thoughtful comment. Unfortunately, we did not ask participants specifically if they were currently enrolled in college. As part of the demographic information we did collect information about education levels (n = 60 reported having some college and the rest, n = 172, had 4 year college degrees or higher and n = 27, had an associates degree). Thus it is possible that the 60 participants who reported having some college education were also currently enrolled in college. This is a limitation and we appreciate the reviewer for helping us see it. We have added language in the discussion section to acknowledge this limitation explicitly, see page 23. We have also changed the title of the manuscript to more accurately reflect the samples used in Study 1 and Study 2. See page 1.***

8. When describing the ACEs, the author may want to align their categories more closely to what the original developer (Feletti) identified them as. Specifically, “criminal behavior in the household” should be changed to “incarcerated household member”, as these insinuate two fairly different experiences
   ***We appreciate the reviewer for catching this error. We replaced “criminal behavior in the household” with “incarcerated household member” in the manuscript.*** ***Please page 9.***
9. Language should be changed to how the analysis was conducted, not their plan for the analysis (written in future tense)

***We thank the reviewer for catching this inconsistency in the language of the manuscript. We fixed the language as recommended. See pages 13-14.***

10. Author states that they used Blom’s rank-based inverse normal transformation on the relevant variables; authors should specific which ones these variables are, or if this was used on all variables

***We appreciate the reviewer for pointing this out. We regret that we were not clear in our writing. We have amended the language to clearly demarcate which variables were transformed using Blom’s method. Please see page 14.***

11. First sentence describes rates for at least 1 ACE and then 3 or more, but college sample is described as 38.6% experiencing ACE’s. Why the difference in reporting?

***We thank the reviewer for catching this inconsistency in reporting the results of Study 1 versus Study 2. We have changed things so the reporting of Study 1 and Study is consistent. Please see page 18.***

12. Author could benefit from looking into “post traumatic growth” literature to check if flexibility in relation to ELA is common; mentioned that there is little research on this subject, but may be more within the subfield of positive psychology

***We thank the reviewer for this feedback. We have added references (e.g. Mittal et al., 2015) from the field of positive psychology to bolster the literature on ELA and cognitive flexibility. Please see pages 20-21.***

13. Perseverative errors is the outcome most frequently reported to measure cognitive flexibility – but this was only significant in adult sample. Should address this more in Discussion.

***We thank the reviewer for this suggestion. We concur with their assessment and have discussed this finding more in the discussion section. Please pages 21-22.***

14. What about total correct not being significant – why not addressed in discussion?

***We appreciate the reviewer for pointing this out. We have added language in the discussion section that addresses the lack of association between total correct and ACEs. Please see page 22.***

15. Address that variance was rather small for regressions.

***We thank the reviewer for pointing this out. We have added language to the discussion as suggested. See page 24.***

16. How meaningful are these results – meaning how far apart were scores on WCST? A significant difference in neuropsych results does not always equate to meaningful functional differences.

***We appreciate the reviewer for pointing this out. We concur with their assessment and have amended the language in the relevant section of the discussion to state that a small difference in neuropsychological scores may not reflect behavioral differences in real life. Please see page 20.***

17. Limitations – note differences between 17 item ACES and 10 item ACES

***We appreciate the reviewer for pointing this out. We have added this as a limitation in the discussion section of the manuscript. Please see page 23.***

18. Spell out how ELA could provide some cognitive benefits.

***We appreciate this comment and have added language in the discussion as suggested that spells out cognitive benefits associated with ELA. Please see page 22.***

Reviewer 2:

1. Minor grammatical errors throughout (e.g., inappropriate use of semi-colons, run on sentences, etc.). For example, the semi-colons on line 27 and on line 78 should be replaced with commas. These grammatical errors are minor and a final review/polish of the manuscript is recommended.

***We thank the reviewer for their keen eye. We have addressed the problems with semi-colons and run on sentences throughout the manuscript. A final review of grammar and punctuation throughout the manuscript was also completed. All errors identified have been corrected.***

2. The methods of data analysis and regression models are well done and nicely presented.
   ***We thank the reviewer for this comment and appreciate their feedback.***
3. It would be interesting to have a bit more discussion on the implications of your findings and what it means for adults who've experienced adverse events as children. For example, how would this reduced ability to shift-focus affect people in their jobs, education, or at home/taking care of a family?

***We thank the reviewer with this suggestion. We have added language to the discussion section as recommended. Please see page 21.***

4. Nice paper that demonstrates a significant finding; as a suggestion for future research, it would be interesting to repeat this in a population that had experienced greater amounts of trauma.

***We thank the reviewer for appreciating our work and for the suggestions of future directions.***
